# Erythritol, Erythronate, and Cardiovascular Outcomes in Older Adults in the ARIC Study

**DOI:** 10.1016/j.jacadv.2025.101605

**Published:** 2025-02-20

**Authors:** Layla A. Abushamat, Bing Yu, Ron C. Hoogeveen, Caroline Sun, Chao Cheng, Sean M. Hartig, Mark A. Herman, Ashok Balasubramanyam, Jane EB. Reusch, Elizabeth Selvin, Chiadi E. Ndumele, Vijay Nambi, Christie M. Ballantyne

**Affiliations:** a Section of Cardiovascular Research, Department of Medicine, Baylor College of Medicine, Houston, Texas, USA; b Department of Epidemiology, School of Public Health, University of Texas Health Science Center at Houston, Houston, Texas, USA; c Section of Epidemiology and Population Science, Department of Medicine, Baylor College of Medicine, Houston, Texas, USA; d Section of Endocrinology, Diabetes and Metabolism, Department of Medicine, Baylor College of Medicine, Houston, Texas, USA; e Division of Endocrinology, Metabolism, and Diabetes, Department of Medicine, University of Colorado Anschutz Medical Campus, Aurora, Colorado, USA; f Rocky Mountain Regional VAMC, Aurora, Colorado, USA; g Department of Epidemiology, Johns Hopkins Bloomberg School of Public Health, Baltimore, Maryland, USA; h Johns Hopkins Ciccarone Center for the Prevention of Cardiovascular Disease, Johns Hopkins University School of Medicine, Baltimore, Maryland, USA; i Section of Cardiology, Department of Medicine, Baylor College of Medicine, Houston, Texas, USA

**Keywords:** artificial sweetener, coronary heart disease (CHD), heart failure, obesity, older adults, stroke, type 2 diabetes

## Abstract

**Background:**

Circulating erythritol, an endogenously produced metabolite and an artificial sweetener, is associated with cardiovascular outcomes.

**Objectives:**

The authors assessed associations of erythritol and its downstream metabolite, erythronate, with cardiovascular risk factors and events in older adults in the ARIC (Atherosclerosis Risk In Communities) study (visit 5, 2011-2013).

**Methods:**

We included 4,006 participants without prevalent cardiovascular disease and with metabolomic profiling. Erythritol and erythronate were measured by mass spectrometry. We analyzed associations of log-transformed erythritol and erythronate with cardiovascular risk factors and events using Cox proportional hazard models.

**Results:**

Participants in the highest tertiles of erythritol or erythronate were older, more likely to have diabetes, hypertension, hyperlipidemia, or microalbuminuria, and had higher body mass index and cardiac biomarkers and lower estimated glomerular filtration rate (*P* < 0.001). Over median follow-up of 8.41 (7.62, 8.93) years, higher erythritol and erythronate concentrations were significantly associated with heart failure (HF) hospitalization, HF with preserved ejection fraction, cardiovascular death, and total mortality after adjustment for demographics and traditional cardiovascular risk factors. Erythronate was additionally significantly associated with coronary heart disease (HR: 1.30 [95% CI: 1.04-1.61], *P* = 0.02), stroke (1.40 [95% CI: 1.08-1.83], *P* = 0.012), and HF with reduced ejection fraction (1.38 [95% CI: 1.09-1.74], *P* = 0.007). Diabetes status did not modify any of these associations (*P* for interaction >0.20).

**Conclusions:**

Circulating erythritol and erythronate levels are markers of cardiometabolic health and cardiovascular outcomes in an older adult population. In particular, erythronate is associated with all cardiovascular outcomes assessed. Future studies should assess the role of erythronate and its related pathways in cardiovascular disease.

The incidence and prevalence of obesity and type 2 diabetes have both increased substantially over the last 2 decades.^1^ People with obesity and/or diabetes have a high risk of atherosclerotic cardiovascular disease (CVD) and heart failure (HF). Additionally, cardiac–kidney–metabolic syndrome is recognized as a leading cause of increased cardiovascular morbidity and mortality. Because of public health emphasis on reducing dietary added sugars to improve cardiometabolic health,^2^ artificial sweeteners are commonly used as an alternative to added sugar by people with overweight or obesity, diabetes, and other cardiometabolic risk factors.^3^ Erythritol was introduced into the U.S. food supply in the 1990s and approved by the U.S. Food and Drug Administration in 2002 as a low-calorie artificial sweetener. Along with excellent tolerability and short-term risk profile, erythritol, a naturally occurring sugar alcohol, was touted as “natural”.^4^ However, artificial sweetener use, including erythritol, is linked to increased coronary heart disease (CHD),^5^ and the long-term safety of erythritol and other artificial sweeteners remains unclear.

Metabolomic analysis of ARIC (Atherosclerosis Risk In Communities) study samples collected in 1987 to 1989 (visit 1) linked erythritol to CHD.^6^ In this study, the association with CHD persisted after adjusting for body mass index (BMI).^6^ Notably, this study was conducted prior to erythritol’s introduction into the U.S. food supply, suggesting a link between endogenously produced erythritol and CHD. It is known that circulating erythritol is metabolized to erythronate via the pentose phosphate pathway (PPP).^7^

The goal of this study was to identify characteristics associated with elevated erythritol and its downstream metabolite, erythronate, and to investigate the association of erythritol and erythronate with future cardiovascular outcomes in older adults at ARIC visit 5 (2011-2013). We hypothesized that elevated erythritol and erythronate are associated with cardiovascular risk factors and, therefore, increased risk of future cardiovascular events.

## Methods

### Study population

ARIC^8^ is a prospective observational study of CVD incidence in adults recruited from 4 U.S. communities. The Institutional Review Boards of all participating centers approved the study protocol; all participants provided written informed consent. ARIC visit 5 (2011-2013) was the index visit for this analysis. Participants excluded from the analysis included those with prevalent CHD, stroke, or HF; race (self-reported) other than White or Black and Black participants at the Minneapolis or Washington field centers because of small numbers; and missing data (Supplemental Figure 1). Prevalent CHD, stroke, and HF were defined as previously reported.^9^

### Metabolite analysis

Metabolomic profiling was performed on visit 5 serum samples (stored at −80 °C since collection) by Metabolon Inc (Durham, NC) using untargeted, liquid chromatography/mass spectrometry–based quantification protocol.^10^ Identification of metabolites was performed as described previously.^11^ Metabolite levels were winsorized at the 1st and 99th percentile (with missing/below-detection-limit values given the lowest detected value of that metabolite) and standardized prior to analysis. Erythritol and erythronate were 2 of 790 metabolites measured. The natural log-transformed metabolites, including erythritol and erythronate, were normalized by their means and standard deviations prior to the analysis.

### Outcomes

Primary outcomes were incident CHD, stroke, HF hospitalization, CVD mortality, and total mortality after the index visit. HF was further adjudicated as HF with preserved ejection fraction (HFpEF: left ventricular ejection fraction ≥50%) or HF with reduced ejection fraction (HFrEF: left ventricular ejection fraction <50%). Incident CHD events encompassed fatal CHD and definite or probable myocardial infarction. Incident stroke events included ischemic strokes and definite or probable hospitalized embolic or thrombotic strokes. CVD deaths were ascertained from hospital discharge records and death certificates. The cutoff date for administrative censoring for events was December 31, 2020.

### Statistical analysis

Log-transformed and standardized erythritol and erythronate relative concentrations were evaluated in categorical (tertiles) and continuous analyses. The relationships between erythritol and erythronate at visit 5 and incident HF hospitalization, CHD, stroke, and CVD death after visit 5 were initially explored using restricted cubic splines adjusted for age, race, and sex. Associations between tertiles of erythritol and erythronate and accumulated incidence of cardiovascular outcomes were assessed by Kaplan–Meier curves. Associations of continuous concentrations of erythritol and erythronate and cardiovascular outcomes were assessed by Cox proportional hazard models. Model 1 adjusted for age, sex, and race-center. Model 2 additionally adjusted for total cholesterol, HDL cholesterol, current smoking, systolic blood pressure, antihypertensive medication use, diabetes status, BMI, estimated glomerular filtration rate, lipid-lowering medication use, and high-sensitivity C-reactive protein. Because of the high death rate after visit 5, we performed sensitivity analysis evaluating risk for incident CVD events competing with nonevent death. To assess the proportional hazards assumption of the model, graphical examination of log(-log[survival]) vs log(survival time) was used to confirm the curves were roughly parallel.

## Results

### Baseline characteristics and association with cardiovascular risk factors

Of 6,538 participants at visit 5, after exclusions, 4,006 participants were included for this analysis (Supplemental Figure 1). The mean age of the participants was 75.3 ± 5.11 years. Baseline characteristics of the cohort, including cardiovascular risk factors, are presented in Table 1. Distribution of natural log–transformed erythritol and erythronate are shown in Supplemental Figures 2 and 3, respectively. Erythritol and erythronate levels were positively correlated (Pearson correlation coefficient = 0.711, *P* < 0.001).

**Table 1:** Baseline Characteristics of Total Cohort and by Erythritol and Erythronate Tertiles

	Total Cohort	Erythritol [SD of Natural Log]				Erythronate [SD of Natural Log]			
	Overall (N = 4,006)	1st Tertile [−4.013, −0.4863] (n = 1,336)	2nd Tertile [−0.4864, 0.0460] (n = 1,335)	3rd Tertile [0.0467, 5.092] (n = 1,335)	*P* Value	1st Tertile [−4.155, −0.5617] (n = 1,336)	2nd Tertile [−0.5615, 0.1110] (n = 1,335)	3rd Tertile [0.1114, 3.164] (n = 1,335)	*P* Value
Age, y	75.3 ± 5.11	73.9 ± 4.56	75.5 ± 5.01	76.5 ± 5.39	<0.001	73.9 ± 4.56	75.3 ± 4.94	76.7 ± 5.41	<0.001
Female, %	61.31	59.36	61.27	63.30	0.037	62.57	62.32	59.03	0.060
Black, %	19.45	25.37	16.55	16.40	<0.001	27.10	16.10	15.13	<0.001
BMI, kg/m^2^	28.5 ± 5.48	28.0 ± 5.35	28.2 ± 5.18	29.2 ± 5.82	<0.001	27.7 ± 5.23	28.3 ± 5.38	29.4 ± 5.70	<0.001
SBP, mm Hg	130.1 ± 17.53	129.6 ± 17.04	129.8 ± 16.81	130.9 ± 18.68	0.077	130.4 ± 17.11	129.8 ± 17.25	130.1 ± 18.22	0.524
DBP, mm Hg	66.8 ± 10.39	67.7 ± 10.27	66.7 ± 10.11	66.0 ± 10.73	<0.001	67.9 ± 10.36	66.9 ± 10.11	65.6 ± 10.60	<0.001
Pulse pressure, mm Hg	63.3 ± 14.09	61.9 ± 13.12	63.1 ± 13.70	64.9 ± 15.22	<0.001	62.5 ± 13.25	63.0 ± 13.94	64.4 ± 14.96	<0.001
Heart rate, beats/min	65.5 ± 10.88	65.5 ± 10.44	65.5 ± 10.87	65.5 ± 11.32	0.625	65.1 ± 10.30	65.7 ± 11.00	65.8 ± 11.31	0.268
Antihypertensive medication use, %	69.70	61.44	66.29	81.38	<0.001	61.07	67.19	80.86	<0.001
Hypertension, %	70.30	62.69	68.96	79.26	<0.001	63.29	69.16	78.47	<0.001
Fasting glucose, mg/dL	112.0 ± 27.00	107.4 ± 19.30	110.6 ± 24.20	118.2 ± 34.34	<0.001	107.1 ± 20.02	110.6 ± 23.08	118.4 ± 34.58	<0.001
Diabetes, %	29.05	21.35	26.34	39.64	<0.001	20.84	25.40	41.07	<0.001
Current smoking, %	5.85	6.88	5.65	4.98	0.041	5.81	6.15	5.59	0.814
Total cholesterol, mg/dL	185.9 ± 40.44	189.7 ± 39.75	187.4 ± 40.30	180.8 ± 40.77	<0.001	189.9 ± 39.34	188.6 ± 39.78	179.2 ± 41.35	<0.001
HDL-C, mg/dL	53.2 ± 14.04	55.9 ± 14.51	53.2 ± 13.73	50.4 ± 13.31	<0.001	56.9 ± 14.46	53.7 ± 13.92	48.9 ± 12.51	<0.001
LDL-C, mg/dL	107.7 ± 33.38	111.7 ± 33.13	108.7 ± 32.86	102.6 ± 33.53	<0.001	111.4 ± 32.66	110.0 ± 32.81	101.5 ± 33.83	<0.001
Non-HDL-C, mg/dL	132.8 ± 36.32	133.8 ± 35.27	134.2 ± 36.89	130.4 ± 36.68	0.001	133.1 ± 34.86	135.0 ± 35.93	130.3 ± 37.97	0.002
Triglycerides, mg/dL	112 (85, 150)	100 (77, 131)	114 (85, 149)	122 (94, 168)	<0.001	98 (76, 126)	112 (86, 149)	127 (97, 172)	<0.001
Statin user, %	46.94	42.44	45.67	52.71	<0.001	43.49	44.74	52.60	<0.001
Lipid-lowering medication use, %	50.85	45.52	49.59	57.45	<0.001	47.25	48.35	56.97	<0.001
eGFR, mL/min/1.73 m^2^	70.8 ± 16.40	81.7 ± 11.53	71.8 ± 12.27	58.9 ± 16.24	<0.001	82.4 ± 11.08	72.4 ± 11.89	57.6 ± 15.18	<0.001
hs-CRP, mg/L	1.96 (0.96, 4.16)	1.86 (0.85, 3.86)	1.87 (0.92, 4.19)	2.20 (1.09, 4.36)	<0.001	1.68 (0.76, 3.78)	1.88 (0.96, 4.08)	2.36 (1.17, 4.70)	<0.001
NT-proBNP, pg/mL	119.6 (63.2, 226.6)	92.0 (51.7, 169.0)	113.2 (63.1, 215.5)	157.5 (81.6, 313.6)	<0.001	91.4 (52.2, 168.0)	109.3 (60.4, 210.3)	164.6 (86.6, 315.0)	<0.001
hs-TnT, ng/L	10 (7, 15)	8 (6, 12)	10 (7, 14)	12 (8, 19)	<0.001	8 (6, 12)	9 (6, 14)	12 (8, 19)	<0.001
hs-TnI, ng/L	2.9 (2.0, 4.6)	2.6 (1.8, 4.0)	2.8 (1.9, 4.2)	3.6 (2.3, 5.8)	<0.001	2.5 (1.8, 3.9)	2.7 (1.9, 4.2)	3.7 (2.5, 6.0)	<0.001
Adiponectin, μg/mL	10.3 (6.8, 15.5)	10.6 (7.0, 15.5)	10.4 (6.8, 15.3)	9.9 (6.7, 15.5)	0.149	11.0 (7.3, 16.6)	10.4 (7.0, 15.4)	9.7 (6.4, 14.7)	<0.001

BMI = body mass index; DBP = diastolic blood pressure; eGFR = estimated glomerular filtration rate; HDL-C = HDL cholesterol; hs-CRP = high-sensitivity C-reactive protein; hs-TnI = high-sensitivity troponin I; hs-TnT = high-sensitivity troponin T; LDL-C = LDL cholesterol; NT-proBNP = N-terminal pro–B-type natriuretic peptide; SBP = systolic blood pressure.

Values are mean ± SD, median (25th, 75th percentile), or %. *P* trends were calculated by sum of ranks trend test across ordered groups.

Table 1 also displays characteristics associated with tertiles of erythritol and erythronate concentrations. Participants in the highest tertile of relative erythritol or erythronate concentrations were significantly older and more likely to be White. They also were more likely to have cardiovascular risk factors, including diabetes, hypertension, hyperlipidemia, higher BMI, lower HDL cholesterol, lower estimated glomerular filtration rate, microalbuminuria, higher high-sensitivity C-reactive protein, higher N-terminal pro–B-type natriuretic peptide, and higher troponin T and troponin I levels. Relative mean erythritol concentrations were higher in participants with obesity and diabetes compared with lean or obese participants without diabetes (Supplemental Table 1).

### Association with cardiovascular outcomes

**Figure 1:**
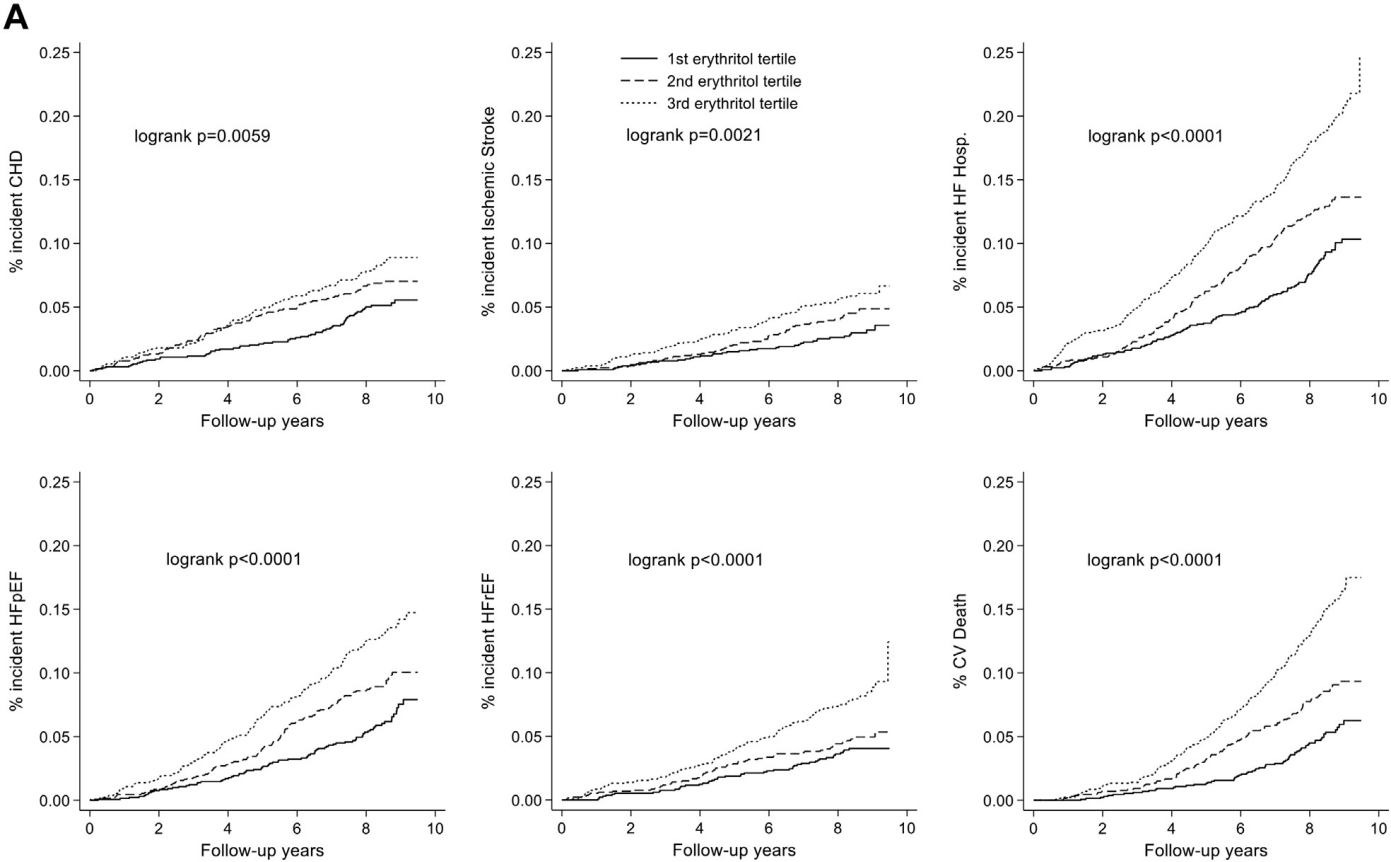

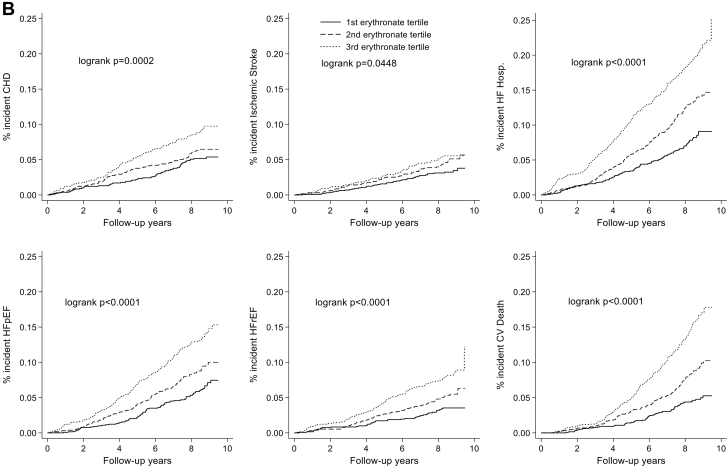
Cumulative Incidence of Cardiovascular Events Across Tertiles of Erythritol and Erythronate Cumulative Incidence of Cardiovascular events across tertiles of (A) erythritol and (B) erythronate. Median (25th percentile, 75th percentile) follow-up was 8.41 (7.62, 8.93) years. The model was adjusted for age, sex, and race (model 1). CHD = coronary heart disease; CV = ccardiovascular; HF = heart failure; HFpEF = HF with preserved ejection fraction; HFrEF = HF with reduced ejection fraction; hosp = hospitalizationa.

Age-,sex-, and race-adjusted models of the associations of erythritol and erythronate (modeled as restricted cubic splines) with incident CHD, stroke, HF hospitalization, HFrEF, HFpEF, CVD death, and total mortality are shown in Supplemental Figure 4. There was a variable association in restricted cubic splines of erythritol with risk of future cardiovascular events. Erythronate had a positive association with risk of future cardiovascular events, particularly at levels above the median. Over a median follow-up of 8.41 (7.62, 8.93) years, there was a significantly greater accumulation of incident cardiovascular events at higher tertiles of erythritol and erythronate (Figures 1A and 1B). When analyzed as a continuous variable, higher erythritol levels at visit 5 were associated with increased risk of HF hospitalization (HR: 1.18 [95% CI: 1.07-1.30], *P* < 0.001), HFpEF (1.16 [95% CI: 1.04-1.31], *P* < 0.009), CVD death (1.21 [95% CI: 1.08-1.35], *P* = 0.001), and total mortality (1.19 [95% CI: 1.12-1.28], *P* < 0.001), whereas higher erythronate levels were associated with CHD (1.30 [95% CI: 1.04-1.61] *P* = 0.02), ischemic stroke (1.40 [95% CI: 1.08-1.83], *P* = 0.012), HF hospitalization (1.53 [95% CI: 1.32-1.79], *P* < 0.001), HFpEF (1.49 [95% CI: 1.24-1.79], *P* < 0.001), HFrEF (1.38 [95% CI: 1.09-1.74], *P* = 0.007), CVD death (1.79 [95% CI: 1.50-2.14], *P* < 0.001), and total mortality (1.66 [95% CI: 1.49-1.84], *P* < 0.001) (Table 2).

**Table 2:** Association of Erythritol and Erythronate at ARIC Visit 5 With Incident CVD Events After ARIC Visit 5

Incident Event	Erythritol		Erythronate	
	HR (95% CI)	*P* Value	HR (95% CI)	*P* Value
CHD (240/4,006, 5.99%)	1.01 (0.85-1.19)	0.925	1.32 (1.06-1.63)	0.012
Ischemic stroke (156/4,006, 3.89%)	1.04 (0.86-1.26)	0.651	1.37 (1.05-1.78)	0.022
HF hospitalization (487/4,006, 12.16%)	1.17 (1.06-1.28)	0.002	1.50 (1.28-1.74)	<0.001
HFpEF (336/4,006, 8.39%)	1.15 (1.03-1.29)	0.016	1.45 (1.20-1.74)	<0.001
HFrEF (196/4,006, 4.89%)	1.09 (0.92-1.29)	0.308	1.33 (1.05-1.68)	0.018
CVD death (336/4,006, 8.39%)	1.19 (1.06-1.33)	0.003	1.72 (1.43-2.06)	<0.001
Total mortality (989/4,006, 24.69%)	1.18 (1.10-1.26)	<0.001	1.61 (1.44-1.79)	<0.001
Non-CVD death (653/4,006, 16.30%)	1.17 (1.07-1.27)	<0.001	1.54 (1.35-1.77)	<0.001

Follow-up for incident CVD events from the date of visit 5 to December 31, 2020. Mean follow-up is 7.7 ± 2.01 years; median (25th, 75th percentile) follow-up is 8.42 (7.62, 8.94) years. Erythritol and erythronate (both natural log transformed) were analyzed as continuous variables. Model adjusted by age, sex, race-center, total cholesterol, HDL-C, current smoking, systolic blood pressure, antihypertensive medication use, diabetes status, BMI, eGFR, lipid-lowering medication use, and log–hs-CRP.

ARIC = Atherosclerosis Risk in Communities; CHD = coronary heart disease; CVD = cardiovascular disease; HF = heart failure; HFpEF = HF with preserved ejection fraction; HFrEF = HF with reduced ejection fraction.

In analysis by diabetes status, participants with diabetes had the same associations of erythritol and erythonate with cardiovascular outcomes as the overall cohort. However, in participants without diabetes, erythritol was associated only with total mortality, and erythronate was associated with HF hospitalization, HFpEF, HFrEF, CVD death, and total mortality (Supplemental Table 2). Despite these differences between the diabetes and no diabetes groups, there was not a significant interaction among these associations by diabetes status (Supplemental Table 3).

## Discussion

Erythritol (C_4_H_10_O_4_) is a 4-carbon sugar alcohol that is 60% to 80% as sweet as sucrose, occurs naturally in soy, some fruits (watermelon, pears, and grapes), and certain alcoholic beverages (wine and sake), and is also used as a non-nutritive, low-calorie sweetener.^12^ Based on 2013 to 2014 National Health and Nutrition Examination Survey data, estimated daily mean intake of erythritol is 32.1 g per day with the highest amount of erythritol, outside of artificial sweeteners, estimated to be in baked goods and fruit snacks.^13^ Increased erythritol levels are due to either exogenous intake or endogenous production and are further metabolized to erythronate. Previous studies of erythritol metabolism after ingestion showed rapid absorption in the gastrointestinal tract with peak plasma levels at 60 to 90 minutes, 60% to 85% urinary excretion within 24 hours after ingestion, and around 90% urinary excretion at 48 hours. Erythritol ingestion does not influence glucose or insulin levels.^12^^,^^14^, ^15^, ^16^, ^17^ Hootman et al^7^ found that 5% to 10% of erythritol is metabolized to erythronate via the PPP.

In this analysis of visit 5 of the ARIC study, erythritol and erythronate were associated with a higher prevalence of cardiovascular risk factors and future cardiovascular outcomes. Participants with diabetes had higher concentrations of erythritol and erythronate compared with participants without diabetes. Higher erythritol and erythronate levels were associated with HF hospitalization, HFpEF, CVD death, and total mortality. Higher erythronate levels were also associated with CHD, ischemic stroke, and HFrEF. The association between erythritol and erythronate and cardiovascular outcomes had no significant interaction with diabetes status. There was similar prevalence of risk factors in the highest tertiles of erythritol and erythronate; however, higher erythronate levels were more consistently associated with cardiovascular outcomes than were erythritol levels (Central Illustration).

**Central Illustration:**
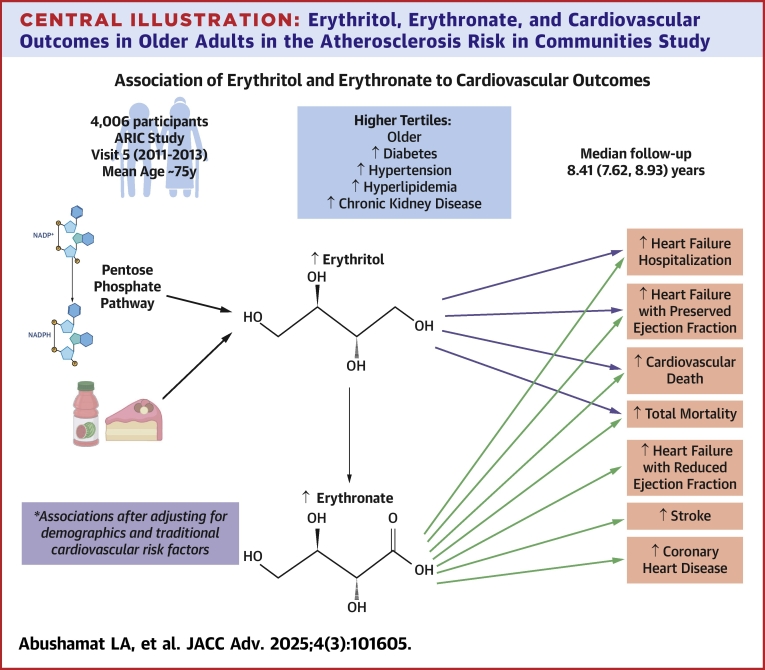
Erythritol, Erythronate, and Cardiovascular Outcomes in Older Adults in the Atherosclerosis Risk in Communities Study Association of erythritol and erythronate to cardiovascular outcomes. Created with BioRender.com.

In epidemiological studies assessing associations of erythritol and health outcomes, it has been difficult to distinguish endogenous production and exogenous intake of erythritol given erythritol’s presence in the food supply. Witkowski et al^18^ recently showed the association of erythritol metabolite levels with increased incident 3-year major adverse cardiovascular events, an association that was confirmed in 2 validation cohorts of patients (median age 62.9-75.0 years) undergoing elective cardiac evaluation. Our analysis similarly found an association of erythritol metabolite levels with cardiovascular outcomes in an older adult population without prior cardiac disease. However, both studies were observational, and erythritol ingestion was not measured or documented.

Similarly, other epidemiological studies support the relationship between erythritol and diabetes as seen in our analysis. Notably, rising erythritol levels correlated with increased diabetes prevalence in all cohorts studied by Witkowski et al^18^ Metabolomic analysis of the TwinsUK cohort showed increased erythritol, classified as a xenobiotic, in people with diabetes and impaired fasting glucose.^19^ In ARIC visit 1 (1987-1989), erythritol was also found to be associated with incident diabetes.^20^ Erythritol production is induced by glucose intake and is associated with weight gain and higher glycemia.^7^ Therefore, individuals who have greater glycemic stress may have increased endogenous production of erythritol, which may merely be a marker of dysglycemia. Although our study found an increased prevalence of diabetes at higher tertiles of erythritol and its metabolite, erythronate, it did not find an interaction between the association of these metabolites and cardiovascular outcomes.

Disentangling the relationship between artificial sweeteners and cardiovascular outcomes is complex because of the inevitable confounding effect of enhanced use of artificial sweeteners in poor–nutritional quality food. Artificial sweetener intake has been shown to be associated with increased CHD and stroke in a large prospective study (mean age 42.22 years, median follow-up 9 years); however, individuals with high artificial sweetener intake also had poorer diet (less fruits, vegetables, and fiber and more processed meat, dairy, and sodium), were less physically active and more likely to smoke, and had a higher BMI, all risk factors for CHD and stroke.^5^ Furthermore, a recent Mendelian randomization study did not find evidence of a causal association between erythritol and diabetes, coronary artery disease, BMI, or chronic kidney disease.^21^ Therefore, these associations may be influenced by confounding factors and reverse causality due to increased artificial sweetener intake in individuals with cardiovascular risk factors and prevalent cardiometabolic disease.

In this analysis, the profile of cardiovascular events associated with erythritol is slightly different than previously reported. In an older population, we observed that higher erythritol was significantly associated only with increased risk of HF hospitalizations, HFpEF, and mortality over a median follow-up of 8.42 (7.62, 8.94) years, in contrast to other studies that showed an association with CHD.^18^ Notably, a more consistent and robust association with all cardiovascular events (including CHD, stroke, and HFrEF) was seen with erythronate than with erythritol. This finding may be due to performing our analysis in an older population without prevalent CHD, stroke, or HF. It may also suggest that the association with cardiovascular outcomes may be due to increased metabolism of erythritol to erythronate endogenously.

We also found that erythritol and erythronate were associated with increased BMI and diabetes prevalence and that individuals with obesity and diabetes had higher relative mean concentrations of erythritol and erythronate compared with lean or obese participants without diabetes. Erythritol has been shown to be endogenously produced from glucose via the nonoxidative portion of the PPP.^7^ The PPP plays an integral role in glucose metabolism, supplying NADPH for fatty acid, nucleotide, sterol, and nonessential amino acid synthesis and ribose-5-phosphate for nucleic acid synthesis. NADPH is also a necessary substrate for both glutathione reduction and production of reactive oxygen species, and therefore, key to redox homeostasis. The nonoxidative portion of the PPP regulates the flux between glycolysis and the oxidative portion of the PPP, with disruption in this balance associated with diabetes, obesity-related inflammation, and diabetic complications.^22^ High erythritol and erythronate may reflect increased flux through the nonoxidative portion of the PPP due to oxidative stress, which is implicated in the progression of cardiometabolic disease.^23^ In vitro studies have shown increased erythritol with chemically induced oxidative stress.^24^ Therefore, the combination of diabetes and obesity could enhance flux through the PPP, enhancing erythronate concentrations.

Erythronate has been previously associated with CVD mortality and all-cause mortality as well as cancer-related mortality in 620 male smokers.^25^ Increased erythronate has been thought to be due to increased flux through the PPP; erythronate is produced as a detoxification product due to upregulated metabolism.^26^^,^^27^ Using lung tumor and cancer cell lines, Zhang et al^26^ recently showed that production of erythronate can also occur via aldehyde dehydrogenase 1 family member A1 in an NAD+-dependent manner. High aldehyde dehydrogenase levels are associated with poor prognosis as a marker of increased tumorigenicity and high-grade tumors.^26^^,^^28^, ^29^, ^30^ Therefore, increased erythronate levels may be a marker of metabolic dysregulation; the unique finding of erythronate’s strong association to all cardiovascular outcomes in this study may reflect overlapping pathophysiological processes of metabolic dysregulation in aging, cancer, and cardiometabolic disease. Further analysis of erythritol and erythronate to assess pathways beyond the PPP may shed further light on these associations and may highlight potential therapeutic targets for these chronic diseases.

Limitations of this analysis include concerns about residual confounding and reverse causality arising from the observational nature of the study. Relative concentrations of erythritol and erythronate were used in the analysis. Metabolomics were performed on fasting serum samples, and variable dietary factors in the 48 hours prior to obtaining the sample can impact these concentrations. Erythritol consumption in this cohort is unknown, and this analysis did not include dietary recall. Furthermore, this analysis was performed on an older adult U.S. population and may not be generalizable to the general population.

Strengths of this analysis include its large, diverse cohort, depth and breadth of data to allow for adjustment of covariates to reduce confounding, and long, extensive follow-up. Based on these results, erythritol and erythronate may serve as biomarkers of poor cardiometabolic health and cardiovascular outcomes in an older adult population without CHD, stroke, or HF.

## Conclusions

In summary, we demonstrated that erythritol and its metabolite, erythronate, were associated with a high burden of cardiovascular risk factors and heightened risk of cardiovascular events in an older population without a history of CVD. Erythronate is associated with all cardiovascular outcomes assessed in this population, suggesting that better understanding of its metabolism should be a future area of research. Future mechanistic studies in humans are also needed to clarify the role of endogenous vs exogenous erythritol in contributing to poor cardiometabolic health.

Perspectives **COMPETENCY IN MEDICAL KNOWLEDGE:** Erythritol, an artificial sweetener that is also produced endogenously in the PPP, has been associated with CHD and CVD. In this study, higher circulating erythritol and its metabolite, erythronate, were associated with higher BMI, hypertension, diabetes, lipid-lowering medication use, and higher levels of cardiac biomarkers (N-terminal pro–B-type natriuretic peptide and troponin). Circulating erythritol and erythronate were associated with incidence of HF and total and CVD mortality. Erythronate was also associated with incidence of CHD and stroke.

**COMPETENCY IN PATIENT CARE:** People with cardiometabolic disease commonly use artificial sweeteners. A better understanding of associations of artificial sweeteners, like erythritol, to cardiovascular outcomes is imperative in this high-risk population.

**TRANSLATIONAL OUTLOOK 1:** In older adults, circulating erythronate was more consistently associated with all cardiovascular outcomes compared with erythritol; erythronate but not erythritol was associated with CHD and stroke, suggesting that erythronate metabolism may shed more light on cardiovascular risk in this population then erythritol alone. Future studies should investigate the role of endogenous vs exogenous erythritol and also include analysis of erythronate levels and related metabolic pathways.

## Funding support and author disclosures

The ARIC study has been funded in whole or in part by Federal funds from the 10.13039/100000050National Heart, Lung, and Blood Institute (NHLBI), 10.13039/100000002National Institutes of Health (NIH), 10.13039/100000016Department of Health and Human Services (DHHS), under Contract nos. (75N92022D00001, 75N92022D00002, 75N92022D00003, 75N92022D00004, 75N92022D00005). Metabolomics measurements were sponsored by the 10.13039/100000051National Human Genome Research Institute (3U01HG004402-02S1). The metabolite measures and Dr Yu were in part supported by R01HL141824. Dr Abushamat is funded by an American Diabetes Association Cardiovascular Metabolism Junior Faculty Award (#4-23-CVD1-01) and a 10.13039/100000002NIH/10.13039/100000062National Institute of Diabetes and Digestive and Kidney Diseases (NIDDK) grant K23DK140641. Dr Selvin was supported by a 10.13039/100000002NIH/10.13039/100000050NHLBI grant K24 HL152440. Reagents for the ALT, AST, GGT, hs-cTnT, and NT-proBNP were donated by the Roche Diagnostics Corporation. Reagents for hs-cTnI were donated by Abbott Diagnostics. Dr Ballantyne has received grant/research support (through his institution) from Abbott Diagnostics, Akcea, Amgen, Arrowhead, Ionis, Lilly, Merck, New Amsterdam, Novartis, and Novo Nordisk and is a consultant for Abbott Diagnostics, Amgen, Arrowhead, AstraZeneca, Denka Seiken, Eli Lilly, Esperion, Illumina, Ionis, Merck, New Amsterdam, Novartis, Novo Nordisk, Roche Diagnostics, and TenSixteen Bio. All other authors have reported that they have no relationships relevant to the contents of this paper to disclose.
