# Supplementary material for: Erythritol, Erythronate, and Cardiovascular Outcomes in Older Adults in the ARIC Study
Source: JACC Adv. 2025 Feb 20;4(3):101605. doi: 10.1016/j.jacadv.2025.101605 (PMC11889355; doi:10.1016/j.jacadv.2025.101605)
Supplement: Supplemental Figures 1, 2, 3, and 4, and Tables 1, 2, and 3 [file mmc1.docx]

**Supplemental Figure 1.** Inclusion/exclusion of participants from ARIC Visit 5 in erythritol/erythronate analysis.

ARIC Visit 5 Cohort (N=6538)

Total Exclusion (N=2532)

<Exclude individuals with race/ethnicity other than African Americans or Whites (N=18)

<Exclude African Americans from Minneapolis or Washington Field Centers (N=24)

< Exclude individuals without metabolite measurements (N=1065)

<Exclude individuals with prevalent CHD (N=889)

<Exclude individuals with prevalent all caused stroke (N=141)

<Exclude individuals with history HF, prevalent HF hospitalization, or missing information on prevalent HF hospitalization (N=395)

Included in ARIC Visit 5 erythritol/erythronate and incident CVD analysis (N=4006)

<1550 male, 2456 female

<3227 White, 779 Black

<Mean age 75.3±5.11 years

**Supplemental Figure 2.** Distribution of ln-erythritol-std. (N=4006).

**Supplemental Figure 3.** Distribution of ln-erythronate-std. (N=4006).

**Supplemental Figure 4.** Cox proportional hazards models of cardiovascular events with (A)Erythritol-std (median =-0.217) and (B) Erythronate-std (median=-0.235) modeled as restricted cubic splines. Knots were placed at the 5th, 27.5th, 50th, 72.5th, and 95th percentiles. The median was used as reference. The model was adjusted for age, sex, and race (model 1). CHD = coronary heart disease; HF = heart failure; hosp=hospitalization; HFrEF = HF with reduced ejection fraction; HFpEF = HF with preserved ejection fraction; CV= cardiovascular.

**A**

**B.**

**Supplemental Table 1.** Relative mean erythritol and erythronate concentrations by groups defined by obesity and diabetes status at ARIC visit 5.

|  | Lean (BMI<25) without diabetes  (N=2022) | Obese (BMI≥30) without diabetes  (N=732) | Obese (BMI ≥30) with diabetes  (N=550) | p-trend |
| --- | --- | --- | --- | --- |
| Erythritol (ln-std) | -0.21 (0.898) | -0.15 (0.941) | 0.19 (1.094) | <0.001 |
| Erythronate (ln-std) | -0.30 (0.832) | -0.20 (0.869) | 0.24 (1.013) | <0.001 |
| *Data presented is mean (ln-std). ln-std=natural logarithm of the standard deviation. p-trend is calculated by test of trend by ordered groups. BMI=body mass index. | | | | |

**Supplemental Table 2. Association of Erythritol and Erythronate and incident events by diabetes status.**

| **Without Diabetes** | | | | | |
| --- | --- | --- | --- | --- | --- |
|  |  | **Erythritol** | | **Erythronate** | |
| **Incident event**  **(event rate)** | **Model** | HR(95% CI) | p-value | HR(95% CI) | p-value |
| **CHD**  **(144/2772, 5.19%)** | **1** | 1.08 (0.91-1.27) | 0.405 | 1.25 (1.04-1.52) | 0.02 |
|  | **2** | 1 (0.80-1.25) | 0.984 | 1.16 (0.86-1.57) | 0.323 |
| **Ischemic stroke**  **(93/2772, 3.35%)** | **1** | 1.05 (0.85-1.29) | 0.679 | 1.22 (0.96-1.56) | 0.106 |
|  | **2** | 0.97 (0.75-1.26) | 0.836 | 1.19 (0.83-1.72) | 0.345 |
| **HF hospitalization (289/2772, 10.43%)** | **1** | 1.18 (1.06-1.31) | 0.002 | 1.41 (1.24-1.61) | <0.001 |
|  | **2** | 1.14 (1.00-1.29) | 0.051 | 1.5 (1.22-1.83) | <0.001 |
| **HFpEF**  **(204/2772, 7.36%)** | **1** | 1.17 (1.03-1.32) | 0.018 | 1.32 (1.13-1.54) | 0.001 |
|  | **2** | 1.13 (0.97-1.32) | 0.112 | 1.31 (1.03-1.67) | 0.03 |
| **HFrEF**  **(104/2772, 3.75%)** | **1** | 1.1 (0.90-1.35) | 0.336 | 1.32 (1.07-1.64) | 0.011 |
|  | **2** | 1.02 (0.78-1.32) | 0.902 | 1.34 (0.97-1.87) | 0.08 |
| **CVD death**  **(187/2772, 6.75%)** | **1** | 1.23 (1.09-1.40) | 0.001 | 1.56 (1.34-1.82) | <0.001 |
|  | **2** | 1.15 (0.98-1.36) | 0.093 | 1.66 (1.30-2.12) | <0.001 |
| **Total mortality**  **(611/2772, 22.04%)** | **1** | 1.19 (1.11-1.28) | <0.001 | 1.44 (1.32-1.57) | <0.001 |
|  | **2** | 1.17 (1.07-1.27) | 0.001 | 1.65 (1.44-1.89) | <0.001 |
| **With Diabetes** | | | | | |
|  |  | **Erythritol** | | **Erythronate** | |
| **Incident event**  **(event rate)** | **Model** | **HR(95% CI)** | **p-value** | **HR(95% CI)** | **p-value** |
| **CHD**  **(92/1135, 8.11%)** | **1** | 1.04 (0.85-1.27) | 0.737 | 1.28 (1.04-1.58) | 0.019 |
|  | **2** | 0.97 (0.75-1.26) | 0.834 | 1.43 (1.04-1.96) | 0.029 |
| **Ischemic stroke**  **(55/1135, 4.85%)** | **1** | 1.09 (0.85-1.39) | 0.5 | 1.25 (0.96-1.62) | 0.1 |
|  | **2** | 1.23 (0.94-1.62) | 0.138 | 1.82 (1.23-2.70) | 0.003 |
| **HF hospitalization**  **(185/1135, 16.30%)** | **1** | 1.32 (1.18-1.48) | <0.001 | 1.59 (1.38-1.83) | <0.001 |
|  | **2** | 1.24 (1.07-1.44) | 0.004 | 1.54 (1.22-1.95) | <0.001 |
| **HFpEF**  **(123/1135, 10.84%)** | **1** | 1.28 (1.12-1.46) | <0.001 | 1.6 (1.34-1.90) | <0.001 |
|  | **2** | 1.22 (1.02-1.46) | 0.026 | 1.74 (1.32-2.31) | <0.001 |
| **HFrEF**  **(90/1135, 7.93%)** | **1** | 1.26 (1.07-1.49) | 0.006 | 1.42 (1.16-1.74) | 0.001 |
|  | **2** | 1.19 (0.96-1.49) | 0.116 | 1.36 (0.97-1.89) | 0.074 |
| **CVD death**  **(135/1135, 11.89%)** | **1** | 1.29 (1.14-1.46) | <0.001 | 1.68 (1.43-1.97) | <0.001 |
|  | **2** | 1.27 (1.08-1.49) | 0.003 | 1.96 (1.50-2.56) | <0.001 |
| **Total mortality**  **(328/1135, 28.90%)** | **1** | 1.21 (1.11-1.32) | <0.001 | 1.4 (1.26-1.56) | <0.001 |
|  | **2** | 1.24 (1.11-1.38) | <0.001 | 1.67 (1.40-1.99) | <0.001 |

Model 1 adjusted for age, sex, and race. Model 2 adjusted for age, sex, race, total cholesterol, HDL-C, current smoking, SBP, antihypertensive medication use, diabetes status, BMI, eGFR, lipid-lowering medication use, and log–hs-CRP. CHD = coronary heart disease; CVD = cardiovascular disease; HF = heart failure; HFpEF = HF with preserved ejection fraction; HFrEF = HF with reduced ejection fraction.

**Supplemental Table 3.** p-value of interaction between erythritol and erythronate and diabetes status

|  | **Erythritol** | | **Erythronate** | |
| --- | --- | --- | --- | --- |
|  | **Model 1** | **Model 2** | **Model 1** | **Model 2** |
| **CHD** | 0.940 | 0.863 | 0.742 | 0.453 |
| **Ischemic stroke** | 0.791 | 0.947 | 0.606 | 0.700 |
| **HF hospitalization** | 0.325 | 0.356 | 0.553 | 0.664 |
| **HFpEF** | 0.582 | 0.599 | 0.299 | 0.320 |
| **HFrEF** | 0.324 | 0.390 | 0.854 | 0.880 |
| **CVD death** | 0.718 | 0.440 | 0.734 | 0.540 |
| **Total mortality** | 0.985 | 0.459 | 0.558 | 0.951 |

Model 1 adjusted for age, sex, and race. Model 2 adjusted for age, sex, race, total cholesterol, HDL-C, current smoking, SBP, antihypertensive medication use, diabetes status, BMI, eGFR, lipid-lowering medication use, and log–hs-CRP. CHD = coronary heart disease; CVD = cardiovascular disease; HF = heart failure; HFpEF = HF with preserved ejection fraction; HFrEF = HF with reduced ejection fraction.
